# Sensitivity of a juvenile subject-specific musculoskeletal model of the ankle joint to the variability of operator-dependent input

**DOI:** 10.1177/0954411917701167

**Published:** 2017-04-21

**Authors:** Iain Hannah, Erica Montefiori, Luca Modenese, Joe Prinold, Marco Viceconti, Claudia Mazzà

**Affiliations:** 1 INSIGNEO Institute for *in silico* Medicine, University of Sheffield, Sheffield, UK; 2 Department of Mechanical Engineering, University of Sheffield, Sheffield, UK

**Keywords:** Repeatability, gait, biomechanics, juvenile idiopathic arthritis, foot, OpenSim, NMSBuilder, magnetic resonance imaging

## Abstract

Subject-specific musculoskeletal modelling is especially useful in the study of juvenile and pathological subjects. However, such methodologies typically require a human operator to identify key landmarks from medical imaging data and are thus affected by unavoidable variability in the parameters defined and subsequent model predictions. The aim of this study was to thus quantify the inter- and intra-operator repeatability of a subject-specific modelling methodology developed for the analysis of subjects with juvenile idiopathic arthritis. Three operators each created subject-specific musculoskeletal foot and ankle models via palpation of bony landmarks, adjustment of geometrical muscle points and definition of joint coordinate systems. These models were then fused to a generic Arnold lower limb model for each of three modelled patients. The repeatability of each modelling operation was found to be comparable to those previously reported for the modelling of healthy, adult subjects. However, the inter-operator repeatability of muscle point definition was significantly greater than intra-operator repeatability (*p* < 0.05) and predicted ankle joint contact forces ranged by up to 24% and 10% of the peak force for the inter- and intra-operator analyses, respectively. Similarly, the maximum inter- and intra-operator variations in muscle force output were 64% and 23% of peak force, respectively. Our results suggest that subject-specific modelling is operator dependent at the foot and ankle, with the definition of muscle geometry the most significant source of output uncertainty. The development of automated procedures to prevent the misplacement of crucial muscle points should therefore be considered a particular priority for those developing subject-specific models.

## Introduction

The use of musculoskeletal models to determine the muscle and joint contact forces (JCFs) during gait has long been reported.^1^ The sensitivity of model outputs to experimental errors such as misplacement of stereophotogrammetric markers and soft tissue artefact has been explored through probabilistic analysis.^2–4^ Similarly, there is a significant body of evidence demonstrating model sensitivity to the defined musculoskeletal anatomy with the joint coordinate systems, inertial parameters, muscle properties and muscle path geometries all investigated.^5–8^ However, the error involved in accurately identifying these anatomical properties from experimental data is less well understood. Due to variability in patient anatomy, concerns have been raised about the accuracy of outputs obtained with scaled, generic models.^9^ This is particularly the case when applying such methods to juvenile or pathological subjects, whose anatomy may differ significantly from the cadavers upon which the generic models are based.^10,11^

Driven by the need for more accurate model predictions and facilitated by advances in medical imaging technology, subject-specific modelling techniques are becoming more widely developed and adopted.^12–19^ One such methodology^20^ was developed for the study of subjects with juvenile idiopathic arthritis (JIA), an autoimmune disease which can cause physical function disabilities as a result of chronic inflammation of the synovial joint membrane. The aetiology of the disease remains unknown but it has been speculated that altered knee and ankle joint loading^21^ may influence disease progression^22^ and is thus a pathology that particularly warrants investigation with subject-specific musculoskeletal models.

As part of such methodologies, analysis of clinical imaging data allows, among other things, subject-specific muscle paths and joint coordinate systems to be identified and defined.^4^ Despite efforts to automate these procedures,^23,24^ this is typically conducted by a human operator and is thus liable to unavoidable inter- and intra-operator variability in the parameters defined.

To justify the time required for an operator to analyse subject medical images and manually modify a model parameter, two criteria should be met: first, that the model outputs are sensitive to its value, and second, that it can be repeatably and reliably identified. As such, several studies have aimed to quantify the variability and sensitivity of the parameters typically defined as part of a subject-specific modelling approach.^25–27^

Martelli et al.^28^ reported the variation in predicted JCFs and muscle forces after altering lower limb joint coordinate systems in line with the inter- and intra-operator distributions. These distributions were determined from those recorded by five operators, each analysing computed tomography (CT) images of a subject. They found the largest impact of operator input on JCFs to be at the ankle, with a maximum change of 0.33 times bodyweight (BW) reported. However, muscle forces were found to vary more significantly, by up to 114% of their median value. Valente et al.^4^ perturbed bony landmark locations, muscle path points and maximum muscle tensions via a Monte Carlo analysis and found them to have a greater impact on the ankle JCFs, with relevant values ranging by up to 1.58 BW, and on muscle forces, which varied by up to 1.58 BW. Such studies are extremely useful, allowing those developing musculoskeletal modelling approaches to identify the subset of critical parameters that are worth varying on a subject-specific basis.

However, the subject-specific models created as part of both of these studies were of healthy adult subjects. Conversely, little research has been done into the repeatability and sensitivity of such modelling methodologies when applied to juvenile or pathological subjects. As such, the aim of the following study was to investigate the inter- and intra-operator repeatability of a subject-specific modelling methodology developed for children with JIA. The sensitivity of the estimated ankle JCFs and muscle forces to the operator-dependent variation in defined muscle geometries and joint coordinate systems was also investigated.

## Methods

### Subjects and data acquisition

The data collection was carried out by specialised clinical centres as part of the MD-Paedigree project (EC 7th FP, ICT Program, CN: 600932). Three female subjects with JIA were selected to take part in the study with written informed consent obtained from all subjects and/or their parents. Subject data, including the number of affected joints, a Child Health Assessment Questionnaire score (CHAQ)^29^ and a composite disease activity score (JADAS-71),^30^ are shown in Table 1. Gait analysis was based on the PlugIn Gait^31^ and modified Oxford Foot Model (mOFM)^32^ marker protocols (for detailed procedures, see Prinold et al.^20^) with three gait trials performed by each subject randomly selected for inclusion in this study.

**Table 1.** Subject data.

	Subject A	Subject B	Subject C
Age (years)	9.5	12.9	15.9
Height (m)	1.37	1.53	1.45
Mass (kg)	40.6	64.2	50.0
BMI (kg/m^2^)	21.5	27.2	23.8
Affected joints	6	5	3
CHAQ	0	0.5	1.75
JADAS-71	13.8	–	16.4

BMI: body mass index.

CHAQ^29^ is a measure of limitation to activities of daily living (range: 0–3; ‘3’ being most severe). JADAS-71^30^ is a composite disease activity score (range: 0–101; ‘101’ being most severe).

Two sequences of magnetic resonance imaging (MRI) scans of the foot and distal tibia were obtained for each subject. The first sequence was a multi-slice, multi-echo 3D Gradient Echo (mFFE) scan in the sagittal plane with a 1 mm slice thickness and 0.5 mm in-plane resolution. The second sequence was a 3D short T1 inversion time inversion recovery fast field echo scan, again in the sagittal plane. The slice thickness was 2 mm with a 0.6 mm in-plane resolution. Subject bony geometries were segmented from the first MRI sequence by a single operator while the data from the second sequence was used to define subject-specific muscle paths.

### Musculoskeletal modelling approach

A generic unilateral lower limb model of each subject was created by scaling the geometry of the Arnold model^33^ with the tools available in OpenSim.^34^ The generic foot was subsequently replaced with a subject-specific, two-segment equivalent, fused to the generic model at the ankle joint. The process to create the subject-specific foot was reported in detail by Prinold et al.,^20^ but is presented in brief here.

Once bony geometries of the foot and distal tibia have been segmented from the imaging data, the process of creating a subject-specific foot model can be broken down into four distinct phases, all of which were performed in NMSBuilder:^4,35^

1. *Virtual palpation of anatomical landmarks.* Key landmarks on the segmented bony geometries were identified by the operator according to Van Sint Jan.^36^ These landmarks were divided into segment landmark clouds with the tibia, hindfoot, talus, metatarsal and forefoot segments requiring 3, 4, 4, 6 and 5 landmarks to be palpated, respectively. The 22 markers virtually palpated in this study are a subset of those reported in Prinold et al.^20^ A full list of the markers used is available as a supplementary file accompanying this article.
2. *Registration of generic muscle atlas.* The location of the virtually palpated landmarks was subsequently used to register a generic atlas of muscle points^33^ on to the subject-specific geometry. These served as first estimate of the subject-specific muscle paths. This process is not operator dependent.
3. *Manual adjustment of muscle paths.* All foot muscle origin, insertion and via points were adjusted by the operator to be consistent with the subject MRI data. Points captured by the MRI scan in the distal tibia were also altered resulting in a total of 74 muscle path points that had to be manually modified.
4. *Definition of joint coordinate systems.* Proximal and distal anatomical coordinate frames were defined for the ankle (tibia–hindfoot) and metatarsophalangeal (MTP) joint (hindfoot–forefoot) via palpation of bony landmarks as in Stebbins et al.^32^ One exception was the ankle joint centre which was determined by fitting a cylinder to the talar dome with its mediolateral axis serving as the plantar/dorsi-flexion axis.^20^

The combined generic lower limb and subject-specific foot model had a total of five segments (pelvis, femur, tibia, hindfoot, forefoot) and 13 degrees of freedom: six at the pelvis, three at the hip, one at the knee (flexion/extension), two at the talocrural ankle joint (inversion/eversion and plantar/dorsi-flexion) and one at the hindfoot–forefoot (plantar/dorsi-flexion). A total of 54 muscle paths were defined in each model, of which 16 span the ankle joint.

### Simulation of gait trials

Muscle forces and JCFs were determined in OpenSim using a standard approach of inverse kinematics, followed by static optimisation and joint reaction analysis.^34^ Model outputs were compared against joint angles, joint moments and muscle activation patterns reported in the literature for level walking.^37–40^ However, no attempts were made to validate the muscle forces obtained with the static optimisation tool against experimentally obtained electromyography measures as this was beyond the scope of the study.

Coordinate actuators were defined at the pelvis while residual actuators were employed at the hip joint only. As a two-segment foot was defined, the single ground reaction force (GRF) as recorded by the force platform had to be divided between the hindfoot and forefoot segments. This was achieved by applying the entire measured load to the hindfoot until the centre of pressure (COP) crossed the metatarsophalangeal joint, at which point the load was applied exclusively to the forefoot segment.^20^

### Operators

Following the methodology described above, a musculoskeletal model of each subject was created by each of three expert operators. One operator completed the full subject-specific modelling approach three times for a single subject (Subject C) such that intra-operator analyses could be performed. A minimum of 48 h was allowed to pass between each intra-operator modelling procedure.

### Statistical analysis

All operator-dependent inputs and model predictions were recorded to allow the robustness of the modelling approach to be investigated. Appropriate statistical tests were selected according to the purpose of the investigation and are detailed hereafter. The level of significance (*p*) was set to be 0.05 in all analyses.

The repeatability of two modelling steps, i.e. the palpation of each virtual landmark and the definition of each muscle point location, was evaluated by calculating the standard deviation (SD) of the spatial coordinates defined for each point. For the analysis of virtually palpated landmarks, each segment landmark cloud was considered to be an independent variable. The repeatability of the definition of the joint coordinate systems was assessed by determining the variability (SD) in the Cardan rotation required to superimpose the proximal frame upon the distal frame for each joint in the model.

**Figure 1.**
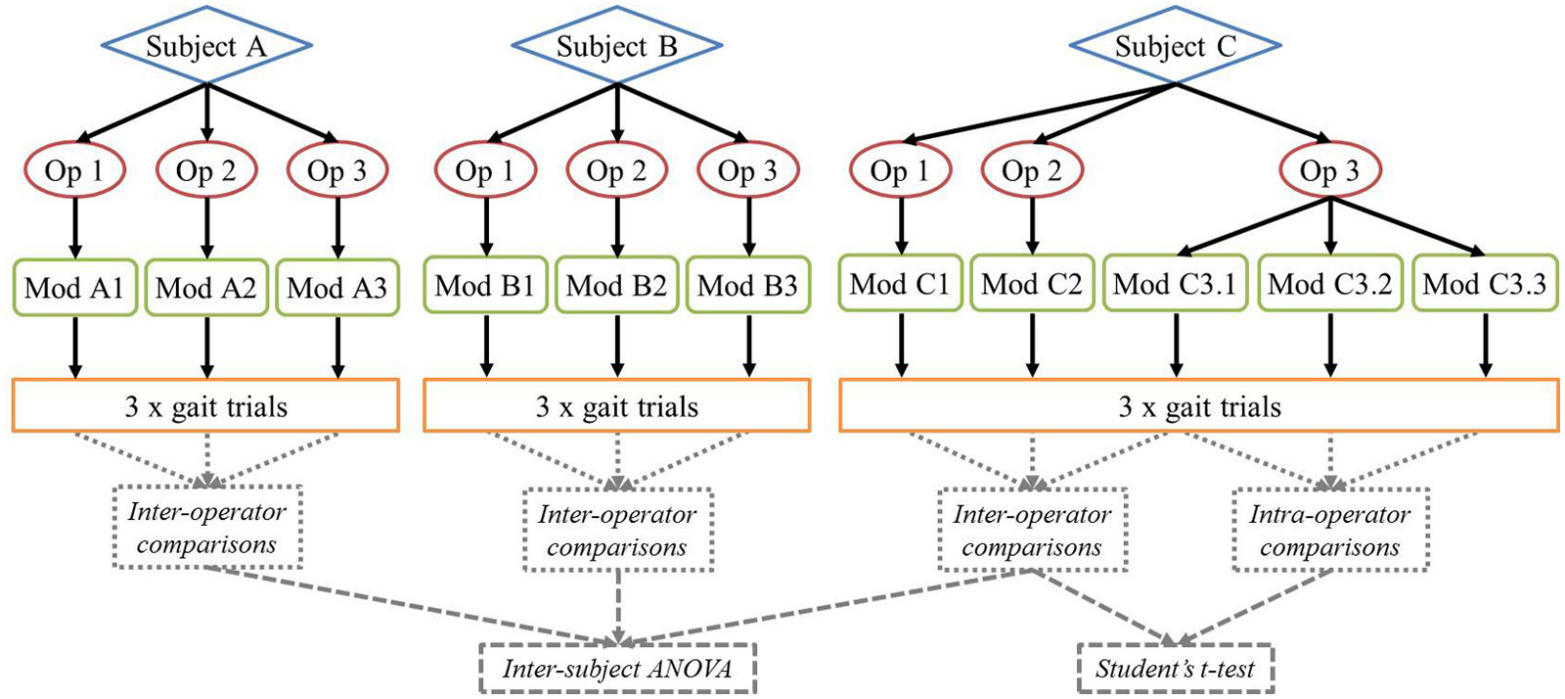
Flow chart illustrating inter- and intra-operator analysis and statistical tests employed. Subjects, operators (Op), models (Mod) and gait trials are shown. Inter- and intra-operator comparisons were performed on both model inputs and outputs.

A one-way analysis of variance was run between the results obtained for each of the three subjects to test whether the anatomy of the patient was a significant factor in the repeatability of the methodology. This was performed at each stage of the modelling process considered (virtual palpation of anatomical landmarks, manual adjustment of muscle paths, definition of joint coordinate systems). Where no statistically significant inter-subject differences were observed, a comparison of inter- and intra-operator repeatability was also performed for one subject (Subject C) using a two-tailed, paired Student’s t-test (Figure 1).

The sensitivity of the ankle JCFs to inter- and intra-operator modelling was assessed via calculation of the variation in the mean vertical ankle JCF predicted for each subject in the ground reference frame across the three simulated gait trials. Similarly, the sensitivity of model estimated muscle forces was investigated by determining the mean of the maximum change in muscle force output at any point during each gait trial. This value was determined for each of six key muscles that cross the ankle joint: soleus, gastrocnemius medialis, gastrocnemius lateralis, tibialis posterior, tibialis anterior and peroneus longus, each of which to whom ankle JCF was shown to be most sensitive in a previous study.^20^ Furthermore, they are also muscles spanning the ankle joint that have the largest physiological cross-sectional area. All JCFs and muscle loads were normalised to subject BW.

## Results

### Variability of model input

The maximum inter-operator SD in defined landmark location were 2.9, 2.9 and 2.7 mm for Subjects A, B and C, respectively, with mean inter-operator repeatability of all virtually palpated landmarks of 0.90 ± 0.60 mm. In comparison, the maximum intra-operator SD was 2.3 mm with a mean across all landmarks of 0.66 ± 0.63 mm. All statistical tests upheld the null hypothesis indicating virtual palpation is both operator and subject independent.

The inter-operator repeatability of the defined muscle point location (3.0 ± 2.5 mm) was found to be significantly lower (*p* < 0.05) than intra-operator repeatability (1.7 ± 1.9 mm) for Subject C. The maximum variation in the spatial dimensions of any single muscle point was 14.3 mm (extensor hallucis brevis – via point) and 9.6 mm (flexor hallucis brevis – origin) for the inter- and intra-operator analyses, respectively.

Mean inter-subject SDs were found to be 3.0 ± 2.9 mm for Subject A, 2.7 ± 2.3 mm for Subject B and 3.0 ± 2.5 mm for Subject C with the maximum SD of a single point being 17.0 mm (flexor hallucis brevis – origin), 12.3 mm (extensor digitorum longus – via point) and 14.3 mm (extensor hallucis brevis – via point), respectively. No significant inter-subject differences were observed. Further analysis of individual muscle points indicated that the forefoot muscle insertion points (flexors and extensors digitorum and hallucis) were the most repeatably identified while operators disagreed more about the location of via points relative to muscle origin and insertion points.

**Figure 2.**
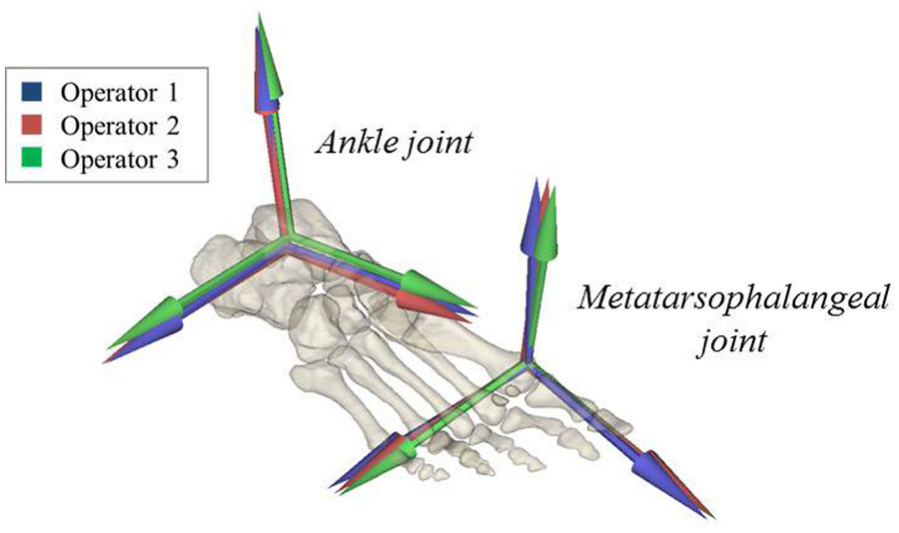
Distal segment anatomical coordinate frames defined by each operator. Ankle and metatarsophalangeal joints (Subject C).

When considering the joint coordinate systems defined in the models (Figure 2), inter-operator SDs were found to range from 1.36° to 3.02° for the ankle inversion/eversion axis and from 0.26° to 1.72° for the plantar/dorsi-flexion axis. Variability at the metatarsophalangeal plantar/dorsi-flexion axis was greater, 2.40°–7.04°. The variance in the intra-operator joint coordinate systems was 0.50°, 1.15° and 0.88° for the three axes, respectively. Inter- and intra-operator repeatability was not found to differ by a statistically significant margin and no inter-subject effects were observed (Table 2).

**Table 2.** Inter- and intra-operator SD (°) in joint angle definitions.

Joint	Inter-operator						Intra-operator	
	Subject A		Subject B		Subject C		Subject C	
	Inv/Ev	PF/DF	Inv/Ev	PF/DF	Inv/Ev	PF/DF	Inv/Ev	PF/DF
	SD (°)	SD (°)	SD (°)	SD (°)	SD (°)	SD (°)	SD (°)	SD (°)
Ankle	1.36	1.64	3.02	1.72	1.36	0.26	0.50	1.15
MTP	–	2.40	–	7.04	–	3.37	–	0.88

SD: standard deviation; MTP: metatarsophalangeal.

Inversion/eversion (Inv/Ev) and plantar/dorsi-flexion (PF/DF) axes are shown.

### Variability of model predictions

**Figure 3.**
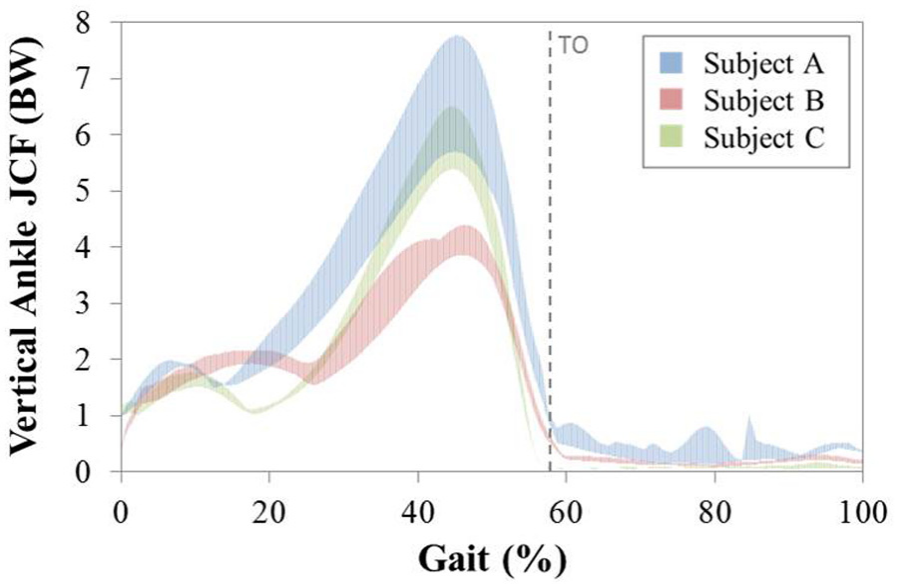
Range of inter-operator mean vertical ankle joint contact forces (BW) obtained across three gait trials in the ground reference frame. Dotted line represents average occurrence of toe-off (TO).

Figure 3 shows the inter-operator variation in the vertical mean ankle JCF calculated for each subject across the three modelled gait trials. The maximum ranges observed were 1.50 BW, 0.75 BW and 0.73 BW for Subjects A, B and C, respectively. The maximum intra-operator range was found to be smaller again, 0.28 BW for Subject C.

The average of the maximum inter-operator changes in vertical ankle JCF observed at any point during a gait trial was 1.55 ± 0.36 BW for Subject A (20% of peak JCF), 0.77 ± 0.31 BW for Subject B (16% of peak JCF) and 0.75 ± 0.02 BW for Subject C (12% of peak JCF) with the maximum recorded in any individual trial of 1.86 BW (Subject A – 24% of peak JCF). The equivalent intra-operator value was smaller, 0.33 ± 0.15 BW (6% of peak JCF), with a single trial maximum of 0.55 BW (10% of peak JCF).

Table 3 shows the average of the maximum difference in estimated muscle force output for six key muscles at any frame in the gait cycle. The muscles with the greatest inter- and intra-operator variation were the soleus, gastrocnemius medialis and tibialis anterior with the differences observed in Subject A consistently larger than with the other two models. The maximum inter-operator difference observed in any one trial was 1.94 BW for Subject A (tibialis anterior – 64% of peak force), 0.96 BW for Subject B (gastrocnemius medialis – 73% of peak force) and 0.94 BW for Subject C (soleus – 40% of peak force). The maximum change output for a muscle force in the intra-operator analysis was 0.44 BW in the soleus (23% of peak force).

**Table 3.** Maximum difference (Max diff) in estimated muscle force.

Muscle	Inter-operator			Intra-operator
	Subject A	Subject B	Subject C	Subject C
	Max diff (BW)	Max diff (BW)	Max diff (BW)	Max diff (BW)
Soleus	1.25 ± 0.09	0.38 ± 0.23	0.85 ± 0.10	0.41 ± 0.02
Gastrocnemius medialis	1.03 ± 0.34	0.47 ± 0.35	0.76 ± 0.06	0.30 ± 0.02
Gastrocnemius lateralis	0.90 ± 0.51	0.31 ± 0.14	0.06 ± 0.00	0.01 ± 0.01
Tibialis posterior	0.98 ± 0.41	0.26 ± 0.08	0.54 ± 0.04	0.01 ± 0.03
Tibialis anterior	1.46 ± 0.29	0.25 ± 0.08	0.19 ± 0.02	0.17 ± 0.02
Peroneus longus	1.03 ± 0.34	0.40 ± 0.25	0.22 ± 0.01	0.08 ± 0.03

BW: bodyweight; SD: standard deviation.

Mean ± SD across three gait trials is shown.

## Discussion

In this study, subject-specific models of three pathological subjects were created such that the inter- and intra-operator uncertainty in model parameter definition could be estimated and the sensitivity of the ankle JCFs and muscle forces output with the models evaluated.

The virtual palpation of bony landmarks was found to be a repeatable operation, both intra- and inter-operator with the mean inter- and intra-operator variation in the defined spatial dimensions of 0.90 and 0.62 mm, respectively. This compares favourably with the value of 1.11 mm reported in a previous experimental study in which five individual operators each palpated subject MRI imagery three times.^4^ However, separate inter- and intra-operator repeatability data were not reported, as here.

The definition of subject-specific muscle paths was found to be subject independent but not operator independent. This is crucial as errors in locating muscular attachments are the largest source of inconsistency in musculoskeletal output.^4,20,23^ The mean variation in muscle point location was 3.0 ± 2.5 mm, lower than the 5.0 mm uncertainty reported by Pal et al.,^41^ when deriving muscle attachment points from the measurement of surface landmarks at the knee, and used as the level of uncertainty in Valente et al.’s^4^ probabilistic analysis. As would be expected, this suggests that the repeatability of identifying muscle paths is improved when an operator has access to medical images of the subject.

Variability in the definition of model joint coordinate systems has been shown to have a minor influence on output JCFs but a considerable impact on the predicted muscle forces.^28^ The mean inter-operator variation in the ankle coordinate systems was 1.2° for the plantar/dorsi-flexion axis and 1.9° for the inversion/eversion axis. These values are comparable to those reported by Martelli et al.,^28^ 0.4° and 2.0°, respectively. Mean variability was higher at the metatarsophalangeal joint, 4.3°, indicating that the bony landmarks used to identify this joint^32^ could be less repeatably identified.

When considering model outputs, the unavoidable variability in operator-defined subject-specific parameter definition had a clear effect on vertical ankle JCFs, with a maximum inter-operator variability of 1.86 BW observed, a value equal to 24% of the peak JCF. This is comparable with a similar study by Valente et al.,^4^ who reported a slightly lower variation of 1.58 BW. However, while both studies varied the location of muscle path points, their study altered the location of bony landmarks and maximum muscle tensions, as opposed to the joint coordinate systems as reported here. Intra-operator variability in ankle JCF was found to be much smaller, only 0.33 BW, indicating that subject-specific model predictions obtained by a single operator are directly comparable. However, these findings can only be said to be valid for vertical ankle JCFs as shear forces have not been considered.

Consistent with previously reported studies,^25,27^ perturbations of model input parameters had a considerable impact on the predicted muscle forces. When varying the defined joint coordinate systems, Martelli et al.^28^ found muscle forces to vary by up to 114% compared to their median value, while Valente et al.^4^ reported a maximum variation of 1.54 BW. These values again compare favourably with the maximum variation in muscle force observed in this study, 1.94 BW. Furthermore, the muscles most affected in Valente et al.^4^ at the ankle (soleus, gastrocnemius medialis, tibialis anterior) are the same as reported here. This indicates that it is the muscles with the larger physiological cross-sectional areas and moment arms that are most affected by uncertainty in their definition, and that their misplacement has the greatest impact on predicted muscle forces and JCFs.^11,20^ Therefore, particular care should be taken when locating their bone insertion and via points.

The estimated inter-operator JCFs and muscle loads were considerably more varied for one subject than the other two. Although no statistically significant inter-subject differences in the model inputs were observed, this subject had the highest levels of variability in the definition of the muscle paths but interestingly, not in the definition of the joint coordinate systems. This is further evidence that it is the spatial location of muscle points which are the greatest source of variability in the outputs obtained with musculoskeletal models.^4,20,23^ As such, the development of appropriate techniques for their reliable identification would be particularly advantageous and enable appropriate muscle moment arms, muscle lines of action and muscle-tendon lengths to be defined.

A number of limitations exist in the reported methodology that should be considered when reviewing the presented results. First, all operators based their models on the same segmented bony geometries, a procedure which, while sometime automated,^42–44^ would also typically entail a further degree of inter-operator variation. The entire modelling methodology was also only completed multiple times by a single operator and for a single subject. While no statistically significant inter-subject differences were observed, the intra-operator analyses presented should therefore be interpreted with an understanding that the inclusion of further subjects and operators in the study could result in differing levels of uncertainty. Furthermore, only the reported subject-specific modelling methodology has been investigated, and adopting an alternative modelling approach may result in differing levels of repeatability and sensitivity.

A further limitation of the study is the use of a static optimisation technique to estimate muscle–tendon forces. Static optimisation assumes that muscle recruitment is such that the metabolic energy expenditure required to facilitate a movement is minimised^45,46^ and this is implemented through the minimisation of an objective function (the sum of muscle activations squared in the case of this study). However, the gait of pathological individuals is likely to be suboptimal with regard to energetic efficiency, instead prioritising the reduction of articular loading at painful joints, for example. Caution should therefore be employed when evaluating the outputs of the model as optimal neuro-motor control has been assumed when simulating the motion of pathological subjects.

Alternative methodological approaches to overcome this limitation, such as personalizing the muscle recruitment strategy using electromyographically driven modelling techniques, are available in the literature.^47^ However, this was not possible as EMG signals for all muscles crossing the ankle joint would be required and these were not collected in this study. Identification of a ‘disease-specific’ objective function would also be a challenging task requiring careful validation and is outside the scope of this investigation.

A final limitation of the reported study is the definition of generic muscle parameters in an otherwise subject-specific foot model, and their subsequent effect on model predictions via the force-length-velocity relationship.^48^ It was considered reasonable to scale optimal fibre lengths and tendon slack lengths such that their relative ratio was maintained with respect to the total muscle-tendon length at rest. However, future studies could determine subject-specific muscle parameters by employing more complex anthropometric scaling tools.^49^ Despite these limitations, it is clear that the reported methodology allowed the stated aim of the study to be achieved, to quantify the sensitivity of a juvenile subject-specific musculoskeletal foot and ankle model to the variation in operator-dependent input.

## Conclusion

This study investigated the inter- and intra-operator repeatability and sensitivity of a subject-specific modelling methodology developed for the analysis of juvenile, pathological subjects. The findings of the study indicate that the reported methodology exhibits comparable levels of repeatability and sensitivity to those reported for modelling healthy adults.^4,28^ Inter-operator variation in the definition of muscle geometries remains significant and has the greatest impact on model outputs. As such, automated routines should be developed to reduce the significance of the operator’s role and prevent the misplacement of crucial muscle points. This will be of particular interest to those developing musculoskeletal models of juvenile or pathological subjects, for whom subject-specific modelling is of the greatest importance.^10,11^
